# Lost in Translation: On the Problem of Data Coding in Penalized Whole Genome Regression with Interactions

**DOI:** 10.1534/g3.118.200961

**Published:** 2019-02-21

**Authors:** Johannes W. R. Martini, Francisco Rosales, Ngoc-Thuy Ha, Johannes Heise, Valentin Wimmer, Thomas Kneib

**Affiliations:** * KWS SAAT SE, Einbeck, Germany; † Universidad del Pacífico, Academic Department of Finance, Lima, Peru; ‡ Department of Animal Breeding and Genetics; ** Chairs of Statistics and Econometrics, University of Goettingen, Goettingen, Germany; § IT Solutions for Animal Production (vit), Verden, Germany

**Keywords:** Interactions, whole genome regression, EGBLUP, GxE, Hadamard products, Genomic selection, Genomic Prediction, GenPred, Shared Data Resources

## Abstract

Mixed models can be considered as a type of penalized regression and are everyday tools in statistical genetics. The standard mixed model for whole genome regression (WGR) is *ridge regression best linear unbiased prediction* (RRBLUP) which is based on an additive marker effect model. Many publications have extended the additive WGR approach by incorporating interactions between loci or between genes and environment. In this context of penalized regressions with interactions, it has been reported that translating the coding of *single nucleotide polymorphisms* -for instance from -1,0,1 to 0,1,2- has an impact on the prediction of genetic values and interaction effects. In this work, we identify the reason for the relevance of variable coding in the general context of penalized polynomial regression. We show that in many cases, predictions of the genetic values are not invariant to translations of the variable coding, with an exception when only the sizes of the coefficients of monomials of highest total degree are penalized. The invariance of RRBLUP can be considered as a special case of this setting, with a polynomial of total degree 1, penalizing additive effects (total degree 1) but not the fixed effect (total degree 0). The extended RRBLUP (eRRBLUP), which includes interactions, is not invariant to translations because it does not only penalize interactions (total degree 2), but also additive effects (total degree 1). This observation implies that translation-invariance can be maintained in a pair-wise epistatic WGR if only interaction effects are penalized, but not the additive effects. In this regard, approaches of pre-selecting loci may not only reduce computation time, but can also help to avoid the variable coding issue. To illustrate the practical relevance, we compare different regressions on a publicly available wheat data set. We show that for an eRRBLUP, the relevance of the marker coding for interaction effect estimates increases with the number of variables included in the model. A biological interpretation of estimated interaction effects may therefore become more difficult. Consequently, comparing *reproducing kernel Hilbert space* (RKHS) approaches to WGR approaches modeling effects explicitly, the supposed advantage of an increased interpretability of the latter may not be real. Our theoretical results are generally valid for penalized regressions, for instance also for the *least absolute shrinkage and selection operator* (LASSO). Moreover, they apply to any type of interaction modeled by products of predictor variables in a penalized regression approach or by Hadamard products of covariance matrices in a mixed model.

Genomic selection based on whole genome regression (WGR) is a crucial ingredient of modern breeding programs (Meuwissen *et al.* 2001; Schaeffer 2006; Habier *et al.* 2007; Hayes *et al.* 2009, 2013; de los Campos *et al.* 2013). The simplest and most successful approach for modeling the genotype-phenotype relation is a linear model assigning an additive effect to each locus (Falconer and Mackay 1996). In more detail, the standard model is given by

$$y=1_{n}\mu +M\beta +\epsilon ,$$ (1)

where y is the n×1 vector of the phenotypic observations of *n* individuals and $1_{n}$ an n×1 vector with each entry equal to 1. Moreover, *μ* is the *y*-intercept, and M the n×p matrix describing the marker states of *n* individuals at *p* loci. Dealing with *single nucleotide polymorphisms* (SNPs) and a diploid species, the entries $M_{i,j}$ can for instance be coded as 0 (aa), 1 (aA or Aa) or 2 (AA) counting the occurrence of the reference allele A. The most frequently used coding subtracts twice the allele frequency, centering each column of M to zero (VanRaden 2008), and alternative approaches consider genotype frequencies (Álvarez-Castro and Carlborg 2007; Vitezica *et al.* 2017). The p×1 vector β represents the allele substitution effects of the *p* loci, and ϵ the n×1 error vector. For single marker regression, which may for instance be used in *genome-wide association studies* (GWAS), we could apply *ordinary least squares regression* (OLS) to estimate $\hat{\beta }$. However, in approaches of genomic selection, we model the effects of many different loci simultaneously and the number of markers *p* is usually much larger than the number of observations *n*. Different methods have been used in the last decades to deal with a large number of variables, among which *ridge regression best linear unbiased prediction* (RRBLUP) is the most popular in quantitative genetics (Schaeffer 2004; Mrode 2014). RRBLUP penalizes the squared $\ell_{2}$ norm of β and has been derived with the additional model specifications of *μ* being a fixed unknown parameter, and $\beta ∼N\left(0,\sigma_{\beta }^{2}I_{p}\right)$ and $\epsilon ∼N\left(0,\sigma_{\epsilon }^{2}I_{n}\right)$ being random. Thus, RRBLUP is not a pure ridge regression penalizing all parameters, but actually a mixed model in which the size of *μ* is not penalized, but only the entries of β are. This mixed model RRBLUP is also called *genomic best linear unbiased prediction* (GBLUP) when Eq.(1) is reformulated with g:=Mβ, and thus $g∼N\left(0,\sigma_{\beta }^{2}{MM}^{t}\right)$. Different variants of ${MM}^{t}$ are called the *genomic relationship matrix* (GRM) (VanRaden 2008). In the additive effect setup, RRBLUP may be considered as the standard reference method (Schaeffer 2004; Mrode 2014), but there are many other approaches which use different assumptions on the effect distribution and other approaches to estimate additive effects (Gianola *et al.* 2009; Gianola 2013).

In particular, because of the immense structural contrast between the statistical additive effect model which does not include any type of interaction between the loci, and biological mechanisms in which interaction is a key concept, scientists have been interested in modeling interaction and “non-additive” genomic relationship (Howard *et al.* 2014). Several manuscripts have addressed the detection of statistical interaction (Cordell 2009; Aschard 2016; Chen *et al.* 2016; Ehrenreich 2017), the role of epistasis in selection response (Carlborg *et al.* 2006; Esfandyari *et al.* 2017; Forneris *et al.* 2017) or the predictive ability of non-additive relationship models. An important class of non-additive relationships is given by *reproducing kernel Hilbert space* models (RKHS) (Gianola and Van Kaam 2008; de los Campos *et al.* 2009; Ober *et al.* 2011; Morota and Gianola 2014; Gianola *et al.* 2014). Moreover, a strongly followed approach simply extends Eq.(1) by explicit dominance effects or by interactions between different loci (Jiang and Reif 2015; Ober *et al.* 2015; Gao *et al.* 2017; Martini *et al.* 2016; Varona *et al.* 2018; Su *et al.* 2012; Xiang *et al.* 2018). The latter approaches have the supposed advantage of being interpreted more easily, since we can estimate interaction effects instead of just dealing with a non-additive genomic relationship model derived from the RKHS setup.

Adding products of predictor variables to model interactions, extends Equation (1) to a polynomial of total degree 2. Whereas subtracting a constant $p_{i}$ from the *i*-th column of M, does neither change the predictions $\hat{y}$ of an OLS regression with interactions (provided it is well-defined) nor those of the penalized regression RRBLUP (provided the penalty factor remains fixed), the predictions of a penalized regression with interactions (*extended RRBLUP* or *eRRBLUP* or *eGBLUP*) are sensitive to a translation of the coding (He and Parida 2016; Martini *et al.* 2017). Moreover, also the estimates of additive effects inferred with RRBLUP or OLS in an additive model are invariant under translations of the variable coding (Strandén and Christensen 2011), but contrary in a model including interactions, the interaction effect estimates will only be unaffected when OLS but not when eRRBLUP is used.

In this work, we address the question why penalized regression is affected by translations of the variable coding when a polynomial model of higher total degree is used. After a theoretical summary of the different methods, we show that in many cases, translating the coding of the predictor variables has an impact on the prediction of genetic values, but that an essential translation-invariant exception is the situation of only penalizing the size of the coefficients of monomials of highest total degree. The invariance of RRBLUP can be considered as a special case of this setting, with a polynomial of total degree 1, where the size of the fixed effect (total degree 0) is not penalized, but only the additive effects (total degree 1) are. The eRRBLUP, which includes interactions, is not invariant to translations because it does not only penalize interactions (total degree 2), but also additive effects (total degree 1). In this regard, approaches of pre-selecting markers, for instance by their additive effect sizes (Kärkkäinen *et al.* 2015), are not only computationally interesting but may also wipe away the coding problem if they allow to model the additive effects as being fixed and to penalize only interactions. Finally, we use a publicly available wheat data set to illustrate that the impact of coding on interaction effect estimates of eRRBLUP becomes stronger with increasing number of variables included in the model. This observation suggests that potential underlying biological interactions of *quantitative trait loci* (QTL) which may be in *linkage disequilibrium* (LD) with the markers, will not only have less influence on estimated interaction effects due to the direct influence of an increasing number of variables across which biological effects may be distributed when fitting the data, but the loss in biological meaning may be enhanced by the secondary effect of an increasing influence of the marker coding. Thus, the supposed advantage of a higher interpretability when modeling interaction effects explicitly in a WGR, compared to RKHS approaches defining non-additive relationships, may only be marginal.

We start with a recapitulation of the regression methods which are relevant for the manuscript.

## Theory: Specification Of Regression Methods

If an expression includes an inverse of a matrix, we implicitly assume that the matrix is invertible for the respective statement, also if not mentioned explicitly. Analogously, some statements for OLS may implicitly assume that a unique estimate exists, which in particular restricts to cases in which the number of observations is at least the same as the number of parameters which have to be determined. Moreover, we will use “estimated” and “predicted” effects as synonym in this work since the quantities may be considered as being fixed or being random in several instances.

### Additive effect regression

The additive effect model has already been presented in Equation (1).

#### OLS:

The ordinary least squares approach determines $\hat{\beta }$ by minimizing the sum of squared residuals (SSR):

$${\left(\begin{array}{c} \hat{\mu }\\ \hat{\beta } \end{array}\right)}_{\text{ OLS}}:={\text{arg min}}_{\left(\mu ,\beta \right)\in {\mathbb{R}}^{p+1}}\sum_{i=1}^{n}{\left(y_{i}-M_{i,•}\beta -\mu \right)}^{2}$$ (2)

$M_{i,•}$ denotes here the *i*-th row of M representing the genomic data of individual *i*. The solution to the minimization problem of Equation (2) is given by the well-known OLS estimate

$${\left(\begin{array}{c} \hat{\mu }\\ \hat{\beta } \end{array}\right)}_{\text{ OLS}}={\left({\left(\begin{array}{cc} 1_{n} & M \end{array}\right)}^{t}\left(\begin{array}{cc} 1_{n} & M \end{array}\right)\right)}^{-1}{\left(\begin{array}{cc} 1_{n} & M \end{array}\right)}^{t}y$$ (3)

provided that the required inverse exists, which in particular also means that *n* has to be greater than *p*.

In problems of statistical genetics, we often deal with a high number of loci and a relatively low number of observations. In this situation of p≥n, the solution to Equation (2) is not unique but a vector subspace of which each point minimizes Equation (2) to zero (“overfitting”). Using an arbitrary value of this subspace, predictions $\hat{y}$ for genotypes which have not been used to estimate the parameters $\left(\hat{\mu },\hat{\beta }\right)$ usually have a low correlation with the corresponding realized phenotypes. An approach to prevent overfitting is RRBLUP.

#### Rrblup / Gblup

minimizes

$${\left(\begin{array}{c} \hat{\mu }\\ \hat{\beta } \end{array}\right)}_{{\text{ RR}}_{\lambda }}:={\text{arg min}}_{\left(\mu ,\beta \right)\in {\mathbb{R}}^{p+1}}\sum_{i=1}^{n}{\left(y_{i}-M_{i,•}\beta -\mu \right)}^{2}+\lambda \sum_{j=1}^{p}\beta_{j}^{2}$$ (4)

for a penalty factor λ>0. Using an approach of maximizing a certain likelihood, the model specifications of $\beta_{j}\overset{i.i.d.}{∼}N\left(0,\sigma_{\beta }^{2}\right)$ and $\epsilon_{i}\overset{i.i.d.}{∼}N\left(0,\sigma_{\epsilon }^{2}\right)$ determine the penalty factor as ratio of the variance components, that is $\lambda :=\frac{\sigma_{\epsilon }^{2}}{\sigma_{\beta }^{2}}$ (Henderson 1975; Henderson and Quaas 1976; Henderson 1977). We stress again that Equation (4) is not a pure ridge regression, as the name RRBLUP might suggest, but a mixed model which treats *μ* and β differently by not penalizing the size of *μ*. This is the version, which is most frequently used in the context of genomic prediction (often with additional fixed effects) (Schaeffer 2004; Mrode 2014).

The corresponding solution is given by

$${\left(\begin{array}{c} \hat{\mu }\\ \hat{\beta } \end{array}\right)}_{{\text{ RR}}_{\lambda }}={\left({\left(\begin{array}{cc} 1_{n} & M \end{array}\right)}^{t}\left(\begin{array}{cc} 1_{n} & M \end{array}\right)+\lambda \left(\begin{array}{cc} 0 & 0_{p}^{t}\\ 0_{p} & I_{p} \end{array}\right)\right)}^{-1}{\left(\begin{array}{cc} 1_{n} & M \end{array}\right)}^{t}y.$$ (5)

where $0_{p}$ denotes the p×1 vector of zeros. The effect of the introduction of the penalization term $\lambda \sum_{j=1}^{p}\beta_{j}^{2}$ is that for the minimization of Equation (4), we have a trade-off between fitting the data optimally and shrinking the squared effects to 0. The method will only “decide” to increase the estimate ${\hat{\beta }}_{j}$, if the gain from improving the fit is greater than the penalized loss generated by the increase of ${\hat{\beta }}_{j}$.

### First order epistasis: Polynomials of total degree two

An extension of the additive model of Equation (1) is a first order epistasis model given by a polynomial of total degree 2 in the marker data (Ober *et al.* 2015; Jiang and Reif 2015; Martini *et al.* 2016)

$$y_{i}=\mu +M_{i,•}\beta +\sum_{k=1}^{p-1}\sum_{j=k+1}^{p}h_{j,k}M_{i,j}M_{i,k}+\epsilon_{i}$$ (6)

Here, all variables are as previously defined and $h_{j,k}$ the interaction effect between loci *j* and *k*. Please note that there is a variant of this model, in which also j=k is included. This interaction of a locus with itself allows to model dominance (Martini *et al.* 2016).

We recapitulate some terms which are important in the context of polynomials in multiple variables. Each product of a subset of the variables $M_{i,1},M_{i,2},\ldots ,M_{i,p}$ is called a monomial. For instance $M_{i,1}$, $M_{i,2}$, $M_{i,1}M_{i,2}$ and $M_{i,1}^{2}$ are four different monomials. Since the product is commutative, $M_{i,1}M_{i,2}$ and $M_{i,2}M_{i,1}$ are the same monomial (and their coefficients are assumed to be summed up in any polynomial which we will address later). The total degree of a monomial is the sum of the powers of the variables in the respective monomial. For instance, $M_{i,1}$ and $M_{i,2}$ are monomials of total degree 1, whereas $M_{i,1}M_{i,2}$, and $M_{i,1}^{2}$ are monomials of total degree 2. Moreover, $M_{i,1}M_{i,2}$ is a monomial of degree 1 in each of the variables $M_{i,1}$ and $M_{i,2}$, and $M_{i,1}^{2}$ is a monomial of degree 2 in $M_{i,1}$ and of degree 0 in $M_{i,2}$. Since a polynomial model is also linear in the coefficients, the regression equations are only slightly modified.

#### Ols:

Equation (3) with a modified matrix M including the products of markers as additional predictor variables represents the OLS solver for model (6).

#### eRRBLUP:

The extended RRBLUP is based on Equation (6) and the assumptions of *μ* being fixed, $\beta_{i}\overset{i.i.d.}{∼}N\left(0,\sigma_{\beta }^{2}\right)$, $h_{j,k}\overset{i.i.d.}{∼}N\left(0,\sigma_{h}^{2}\right)$ and $\epsilon_{i}\overset{i.i.d.}{∼}N\left(0,\sigma_{\epsilon }^{2}\right)$. In this case, the solution is also given by an analog of Equation (5), but with two different penalty factors, $\lambda_{1}:=\frac{\sigma_{\epsilon }^{2}}{\sigma_{\beta }^{2}}$ for additive effects and $\lambda_{2}:=\frac{\sigma_{\epsilon }^{2}}{\sigma_{h}^{2}}$ for interaction effects.

### Translations of the marker coding

In quantitative genetics, column means are often subtracted from the original 0, 1, 2 coding of M to use $\tilde{M}:=M-1_{n}P^{t}$ with P the vector of column means of M (VanRaden 2008) such that

$$\sum_{i=1}^{n}{\tilde{M}}_{i,j}=0\;\forall j=1,\ldots ,p.$$

However, other types of translations, for instance a symmetric {−1,0,1} coding or approaches based on genotypic frequencies (Álvarez-Castro and Carlborg 2007; Vitezica *et al.* 2017) can be found in quantitative genetics’ literature. Thus, the question occurs whether this has an impact on the estimates of the marker effects or on the prediction of genetic values of genotypes.

The answer is that for the additive setup of Equation (1), a shift from M to $\tilde{M}$ will change $\hat{\mu }$ but not $\hat{\beta }$ and any prediction $\hat{y}$ will not be affected, neither for OLS, nor for RRBLUP (provided that λ is not changed) (Strandén and Christensen 2011; Martini *et al.* 2017). This invariance of the additive model does not hold for the extended RRBLUP.

We give an example and discuss the effect of translations of the marker coding in a more general way afterward.

**Example 1** (Translations of the marker coding). *Let the marker data of five individuals with two markers be given*:

$$y={\left(-0.72,2.34,0.08,-0.89,0.86\right)}^{t}M=\left(\begin{array}{cc} 2 & 2\\ 1 & 2\\ 2 & 0\\ 2 & 1\\ 1 & 0 \end{array}\right)$$

*Moreover*, *let us use the original matrix* M, *and the column mean centered matrix* $\tilde{M}:=M-1_{5}\underset{=:P^{t}}{\underset{︸}{\left(1.6,1.0\right)}}$. *We consider the first order epistasis model*

$$y_{i}:=\mu +\beta_{1}M_{i,1}+\beta_{2}M_{i,2}+h_{1,2}M_{i,1}M_{i,2}+\epsilon_{i}$$

*and estimate the corresponding parameters with i) an OLS regression*, *ii) a mixed model regression eRRBLUP-1 with* $\lambda_{1}=\lambda_{2}=1$, *and iii) a mixed model regression eRRBLUP-2 with* $\lambda_{1}=0$ *and* $\lambda_{2}=1$. *The difference between eRRBLUP-1 and eRRBLUP-2 is that the first method penalizes the additive effects and the interaction effect*, *whereas the latter method only penalizes the interaction effect*.

*Let* X *denote the matrix* M *with an additional column of the products of the marker values of each individual*. *Analogously*, $\tilde{X}$ *shall denote the matrix* $\tilde{M}$ *with the additional column of the respective products*.

$$X=\left(\begin{array}{ccc} 2 & 2 & 4\\ 1 & 2 & 2\\ 2 & 0 & 0\\ 2 & 1 & 2\\ 1 & 0 & 0 \end{array}\right)\;and\;\tilde{X}=\left(\begin{array}{ccc} 0.4 & 1 & 0.4\\ -0.6 & 1 & -0.6\\ 0.4 & -1 & -0.4\\ 0.4 & 0 & 0\\ -0.6 & -1 & 0.6 \end{array}\right)$$ (7)

*To estimate the effects with OLS*, *use* Equation (3) *with* X or $\tilde{X}$ *as marker matrix. Analogously*, *use these matrices in Equation (5) for the ridge regression approaches. The difference between eRRBLUP-1 and eRRBLUP-2 manifests only in what is added to the diagonal*:

$${\left(\begin{array}{c} \hat{\mu }\\ {\hat{\beta }}_{1}\\ {\hat{\beta }}_{2}\\ {\hat{h}}_{1,2} \end{array}\right)}_{\;eRRBLUP-1}={\left({\left(\begin{array}{cc} 1_{n} & X \end{array}\right)}^{t}\left(\begin{array}{cc} 1_{n} & X \end{array}\right)+1\cdot \left(\begin{array}{cc} 0 & 0_{3}^{t}\\ 0_{3} & I_{3} \end{array}\right)\right)}^{-1}{\left(\begin{array}{cc} 1_{n} & X \end{array}\right)}^{t}y.$$ (8)

*and*

$${\left(\begin{array}{c} \hat{\mu }\\ {\hat{\beta }}_{1}\\ {\hat{\beta }}_{2}\\ {\hat{h}}_{1,2} \end{array}\right)}_{\;eRRBLUP-2}={\left({\left(\begin{array}{cc} 1_{n} & X \end{array}\right)}^{t}\left(\begin{array}{cc} 1_{n} & X \end{array}\right)+1\cdot \left(\begin{array}{cccc} 0 & 0 & 0 & 0\\ 0 & 0 & 0 & 0\\ 0 & 0 & 0 & 0\\ 0 & 0 & 0 & 1 \end{array}\right)\right)}^{-1}{\left(\begin{array}{cc} 1_{n} & X \end{array}\right)}^{t}y.$$ (9)

*For the centered coding*, *substitute* X *by* $\tilde{X}$. *We summarize our observations from the results presented in* *Table 1* *as follows:*

**Table 1:** **Results from Example 1. “nc” denotes the use of the non-centered matrix** M **and “c” indicates the use of the centered matrix** $\tilde{M}$

	OLS		eRRBLUP-1		eRRBLUP-2	
Estimates	nc	c	nc	c	nc	c
$\hat{\mu }$	1.83	0.33	1.81	0.33	2.69	0.33
${\hat{\beta }}_{1}$	−0.97	−2.11	−0.89	−1.15	−1.54	−2.11
${\hat{\beta }}_{2}$	1.88	0.06	0.71	0.09	1.03	0.11
${\hat{h}}_{1,2}$	−1.14	−1.14	−0.48	−0.57	−0.57	−0.57
						
	−0.91	−0.91	−0.46	−0.27	−0.63	−0.63
	2.34	2.34	1.39	1.46	2.06	2.06
$\hat{y}$	−0.11	−0.11	0.03	0.01	−0.40	−0.40
	−0.51	−0.51	−0.21	−0.13	−0.51	−0.51
	0.86	0.86	0.92	0.59	1.15	1.15

- *Comparing the centered and non-centered versions of OLS*, *the estimates for* μ, $\beta_{1}$ *and* $\beta_{2}$ *change*, *but the estimated interaction* ${\hat{h}}_{1,2}$ *as well as the prediction of* y *remains unchanged*.
- *Comparing the centered and non-centered versions of eRRBLUP-1*, *both codings give different estimates for all the parameters and these solutions produce different predictions for* y.
- *Comparing the centered and non-centered versions of eRRBLUP-2*, *both codings give different estimates for* μ, $\beta_{1}$ and $\beta_{2}$, *but the same for* $h_{1,2}$ *and the same predictions for* y.

The different cases presented in Example 1 have a certain systematic pattern, which we discuss in the following section.

## Theoretical Results

The observations made in Example 1 are explained by the following proposition which has several interesting implications. More formal proofs of the statements made can be found in the Appendix.

**Proposition 1.** *Let* $M_{i,•}$ *be the vector of marker values of individual i and let* $f\left(M_{i,•}\right):{\mathbb{R}}^{p}\to \mathbb{R}$ *be a polynomial of total degree D in the marker data. Moreover, let* $\tilde{M}:=M-1_{n}P^{t}$ *be a translation of the marker coding and let us define a polynomial* $\tilde{f}$ *in the translated variables* $\tilde{M}$ *by* $\tilde{f}\left({\tilde{M}}_{i,•}\right):=f\left({\tilde{M}}_{i,•}+P^{t}\right)=f\left(M_{i,•}\right)$. *Then for any data* y, *the sum of squared residuals (SSR) of both polynomials will be identical (each with the respective coding):*

$$\sum_{i=1,\ldots ,n}{\left(y_{i}-f\left(M_{i,•}\right)\right)}^{2}=\sum_{i=1,\ldots ,n}{\left(y_{i}-\tilde{f}\left({\tilde{M}}_{i,•}\right)\right)}^{2}$$

*Moreover*, *for any monomial m of highest total degree D*, *the corresponding coefficient* $a_{m}$ *of* $f\left(M_{i,•}\right)$ *and* ${\tilde{a}}_{m}$ of $\tilde{f}\left({\tilde{M}}_{i,•}\right)$ *will be identical*:

$$a_{m}={\tilde{a}}_{m}.$$

The coefficients of highest total degree $a_{m}$ represent the additive effects in an addditive effect model, the interaction effects in a model including pair-wise interactions, the three way interactions in a polynomial model of total degree 3, and so on.

The content of Proposition 1 can be summarized the following way: Let us assume that we have data y and a polynomial *f* which is based on marker data M. Moreover, we have the translated data $\tilde{M}$, that is an alternative coding of the predictors. We define the alternative polynomial $\tilde{f}$ by the value of *f* at the corresponding point in the original coding:

$$\tilde{f}\left({\tilde{M}}_{i,•}\right):=f\left({\tilde{M}}_{i,•}+P^{t}\right)=f\left(M_{i,•}\right)$$ (10)

The left hand equation means that we define the alternative polynomial $\tilde{f}$ to be $f\left({\tilde{M}}_{i,•}+P^{t}\right)$ at ${\tilde{M}}_{i,•}$. Then –by definition– the SSR of the fits are identical when each polynomial is used with its respective data coding. Moreover, both polynomials give –by definition– the same predictions $\hat{y}$ to each data point (in its respective coding). Since *f* is given, we have its coefficients (effects) and can thus use the first equality of Equation (10) to calculate the coefficients of $\tilde{f}$. The coefficients of monomials of highest total degree are the same for *f* and $\tilde{f}$. The latter statement needs a little more detailed consideration and we refer to the Appendix. We give an example.

**Example 2**. *In the case of an interaction model based on two loci and without additional restrictions on the coefficients*, *the set of polynomials across which we screen for an optimal fitting one is*

$$\left\{\mu +\beta_{1}M_{i,1}+\beta_{2}M_{i,2}+h_{1,2}M_{i,1}M_{i,2}|\beta_{1},\beta_{2},h_{1,2}\in \mathbb{R}\right\}.$$

*Given the vector* $P^{t}$, *which defines the alternative coding by $\tilde{M}:=M-1_{n}P^{t}$*, *each f can be mapped to an $\tilde{f}$ of Proposition 1 by the left-hand side of Equation (10). This equation states that* $\tilde{f}$, *which is a polynomial in the variables ${\tilde{M}}_{i,•}$, is defined by the original f when we plug in the variables ${\tilde{M}}_{i,•}+P^{t}$ and write down the expression as a function of* ${\tilde{M}}_{i,•}$. *For an example of $P^{t}$ being (0.5,0.3)*, and

$$f=1+2M_{i,1}+0.5M_{i,2}+0.25M_{i,1}M_{i,2},$$

$\tilde{f}$ *would be defined by*

$$\begin{array}{l} \tilde{f}\left({\tilde{M}}_{i,•}\right):=1+2\left({\tilde{M}}_{i,1}+0.5\right)+0.5\left({\tilde{M}}_{i,2}+0.3\right)+\\ +0.25\left({\tilde{M}}_{i,1}+0.5\right)\left({\tilde{M}}_{i,2}+0.3\right) \end{array}$$

*Multiplying the factors gives*

$$\tilde{f}\left({\tilde{M}}_{i,•}\right):=2.1875+2.075{\tilde{M}}_{i,1}+0.625{\tilde{M}}_{i,2}+0.25{\tilde{M}}_{i,1}{\tilde{M}}_{i,2}.$$

*We have calculated $\tilde{f}\left({\tilde{M}}_{i,•}\right)$ which is a function of ${\tilde{M}}_{i,•}$ from the polynomial $f\left(M_{i,•}\right)$ which is a function of $M_{i,•}$. As demonstrated in Proposition 1, both polynomials share the same coefficient for their monomial of highest total degree, that is for $M_{i,1}M_{i,2}$ or ${\tilde{M}}_{i,1}{\tilde{M}}_{i,2}$, respectively. Moreover –due to the way $\tilde{f}$ was constructed from f– all predictions $\hat{y}$ will be identical when the respective coding is used. For instance $f\left(2,2\right)=7=\tilde{f}\left(1.5,1.7\right)$. In particular, this is also true for the data points which are used to estimate the coefficients, and thus the SSR is identical for both polynomials (each with the respective coding).*

Provided that $\tilde{f}$ of Proposition 1 is a valid fit, the statements directly imply that OLS predictions for y are invariant to translations of the coding. The reasoning is the following: If any *f* has a corresponding $\tilde{f}$ which has the same predictions $\hat{y}$ and the same SSR, this is also true for the OLS solution which minimizes the SSR. To make sure that each $\tilde{f}$ is a valid fit, the possibility to adapt coefficients of monomials of lower total degrees is required. We cannot adapt the regression completely if certain coefficients are forced to zero by the model structure. If a coefficient is equal to zero in *f*, it may be different from zero in $\tilde{f}$. We illustrate this with an example.

***Example 3*** *(Models without certain terms of intermediate total degree). Let us consider the data* M *and* y *of Example 1 but with the assumption that marker 2 does not have an additive effect*, *which means that we force the additive effect of marker 2 to zero by the model structure. Then the effect estimates*

$${\left(\begin{array}{c} \hat{\mu }\\ {\hat{\beta }}_{1}\\ {\hat{h}}_{1,2} \end{array}\right)}_{OLS}=\left(\begin{array}{c} 3.710\\ -2.098\\ -0.012 \end{array}\right)\;and\;{\left(\begin{array}{c} \tilde{\mu }\\ {\tilde{\beta }}_{1}\\ {\tilde{h}}_{1,2} \end{array}\right)}_{OLS}=\left(\begin{array}{c} 0.334\\ -2.11\\ -1.162 \end{array}\right)$$

*as well as the estimates* $\hat{y}$ *and* $\tilde{y}$ *are different for both codings*.

Example 3 illustrates a situation in which the OLS is not invariant to the change in the marker coding. The cause of this affectedness is the lack of the additive effect $\beta_{2}$ in the model. Thus, a solution *f* fitting the data for the one coding may lead to an $\tilde{f}$ in which the additive effect of the second marker is non-zero. Thus $\tilde{f}$ is not a valid fit. A certain “completeness” of the model is required to have the possibility to adapt to translations of the coding. We define this property more precisely.

**Definition 1** (Completeness of a polynomial model). *Let* $M_{i,•}$ *be the vector of the marker values of individual* i and let $f\left(M_{i,•}\right):{\mathbb{R}}^{p}\to \mathbb{R}$ *be a polynomial of total degree D in the marker data. The polynomial model f is called complete if for any monomial* $M_{i,j_{1}}^{d_{1}}M_{i,j_{2}}^{d_{2}}\cdots M_{i,j_{m}}^{d_{m}}$ *of f*, *all monomials*

$$M_{i,j_{1}}^{\delta_{1}}M_{i,j_{2}}^{\delta_{2}}\cdots M_{i,j_{m}}^{\delta_{m}}\;\forall \;0\leq \delta_{1}\leq d_{1},\;\forall \;0\leq \delta_{2}\leq d_{2},\;\ldots \;,\forall \;0\leq \delta_{m}\leq d_{m}$$

*are included with a coefficient to be estimated.*

Definition 1 states that for each monomial which is included in the model, all “smaller” monomials have to be included as well. We illustrate this with some examples. Let us consider Equation (6). Its monomials are of shape $M_{i,k}$ or $M_{i,k}M_{i,l}$. For $M_{i,k}$, Definition 1 states that $M_{i,k}^{0}=1$ and $M_{i,k}^{1}$ have to be included, which is obviously the case. For $M_{i,k}M_{i,l}$, $M_{i,k}^{0}=1$, $M_{i,k}^{1}$ and $M_{i,k}^{1}M_{i,l}^{1}$ have to be included, which is also true. Thus, the model is complete. Analogously, if we also include the interactions $M_{i,k}^{2}$, that is if we allow j=k, the model remains complete since all smaller monomials are included. Contrarily, Example 3 is based on the model

$$y_{i}=\mu +\beta_{1}M_{i,1}+h_{1,2}M_{i,1}M_{i,2}+\epsilon_{i}.$$

Since $M_{i,1}M_{i,2}$ is included with a coefficient to be estimated, $M_{i,1}$ and $M_{i,2}$ have to be included to make the model complete. Since $M_{i,2}$ is not included, the polynomial is not complete.

Given that the model is complete and thus allowing an adaption from *f* to $\tilde{f}$, Proposition 1 has various implications. The following corollaries explain the results observed in our examples and highlight some additional properties of penalized regression methods in general. For all statements, it is assumed that penalty factors remain unchanged and that the model is complete.

**Corollary 1.** *For a complete polynomial model of total degree D*, *the OLS estimates of the coefficients of highest total degree as well as the predictions $\hat{y}$ are invariant with respect to translations of the marker coding.*

Corollary 1 is a result of the OLS method being defined only by the SSR, and *f* and the corresponding $\tilde{f}$ of Proposition 1 fitting the data with the same SSR and with the same prediction $\hat{y}$ when their respective coding is used. The statement of Corollary 1 has been observed in Example 1, where the OLS fits for $\hat{y}$ are identical when the coding is translated, and where the estimated coefficients ${\hat{h}}_{1,2}$ of highest total degree remain unchanged.

**Corollary 2.** *For a complete polynomial model of total degree D*, *and a penalized regression which only penalizes the coefficients of monomials of highest total degree*, *the estimates of the coefficients of monomials of highest total degree*, *as well as the predictions $\hat{y}$ are invariant with respect to translations of the marker coding.*

Corollary 2 is a result of the following observation: for each *f*, its corresponding $\tilde{f}$ will have the same SSR (each polynomial with its respective coding), and the same coefficients of highest total degree. Thus, it will have the same value for the target function which we aim to minimize (The target function is the analog of Equation (4) with the corresponding interactions and with a penalty on only the coefficients of monomials of highest total degree). Because this is true for any polynomial *f*, it is in particular true for the solution minimizing the target function. A central point of Corollary 2 is that it is valid for any penalty on the size of the estimated coefficients of highest total degree. The sufficient condition is that only these coefficients of highest total degree are penalized.

**Corollary 3.** *RRBLUP predictions $\hat{y}$ are invariant with respect to translations of the marker coding.*

Corollary 2 applied to complete models of total degree 1 gives the result of Corollary 3, that is RRBLUP being invariant to translations of the marker coding. This fact has been previously proven using a marginal likelihood setup (Strandén and Christensen 2011), or the mixed model equations (Martini *et al.* 2017).

**Corollary 4.** *An additive least absolute shrinkage and selection operator (LASSO) regression (**Tibshirani 1996**) based on a polynomial model of total degree 1 and $\ell_{1}$ penalizing the additive marker effects but not the intercept, is invariant to translations of the marker coding.*

Corollary 4 is a special case of Corollary 2.

Before we illustrate the impact of marker coding on estimated effect sizes with a publicly available data set, we give a small example, highlighting cases which are not invariant to translations of the marker coding.

**Example 4** (Regressions affected by marker coding).

- Whereas, RRBLUP with the fixed intercept is invariant to translations (Strandén and Christensen 2011), pure ridge regression of an additive model of Equation (1) with a penalty on the size of μ (“random intercept”) is not invariant to translations.
- RRBLUP without intercept is not invariant to translations of the marker coding.
- An extended LASSO $\ell_{1}$ penalizing additive effects and interactions is not invariant to translations of the coding.

**Remark 1.** *Proposition 1 stated that the coefficients of monomials of highest total degree D of f and* $\tilde{f}$ *will be identical*. *This statement can even be generalized. Consider for instance the model*

$$\begin{array}{l} y_{i}=f\left(M_{i,1},M_{i,2},M_{i,3}\right)+\epsilon_{i}=\\ =\mu +\beta_{1}M_{i,1}+\beta_{2}M_{i,2}+\beta_{3}M_{i,3}+h_{2,3}M_{i,2}M_{i,3}+\epsilon_{i} \end{array}$$

*The model is a polynomial f of total degree 2. Thus, Proposition 1 states that the coefficient of monomial $M_{i,2}M_{i,3}$ will be identical for f and $\tilde{f}$. However, since $M_{i,1}$ is not included in any other monomial, its coefficient will also be identical for both polynomials. We did not generalize Proposition 1 into this direction to make the manuscript not more technical than necessary. The statement we made in Proposition 1 is sufficient to explain the observations related to genomic prediction models.*

## Practical Implications: An Example With A Wheat Data Set

We illustrated by theoretical considerations and small examples that penalized polynomial regression is in many cases affected by translations of the marker coding. An important exception is the case in which only coefficients of monomials of highest total degree are penalized. To illustrate the differences in estimated effect sizes that may be expected with real data, we compare the estimated interaction effects for different codings on a publicly available wheat data set (Crossa *et al.* 2010). Moreover, we assess the impact of a changed coding on explained variance and out-of-sample predictions.

### Data and method

#### Data:

We use a well investigated wheat data set providing the state of 1279 presence/absence markers of 599 genotyped wheat lines together with records on their yield when grown in four different environments. The yield measurements are standardized to mean 0 and variance 1 (Crossa *et al.* 2010). The provided coding of the marker data are a 0,1 coding. For more details on the data see Crossa *et al.* (2010) or the R (R Core Team 2016) package BGLR (de los Campos and Perez Rodriguez 2016).

#### Codings compared:

We compare three different codings: The originally provided 0,1 coding, a version translated by −0.5, that is a symmetric ±0.5 coding, and a coding in which the mean of each column is subtracted (VanRaden 2008). We refer to these codings as the *original* coding, the *symmetric* coding and the *centered* coding.

#### Interaction effect estimates under different codings and varying number of markers:

The assessment of the practical impact of translations of the marker coding on the effect estimates is difficult. Since in practice, the variance components and consequently the penalty factors are estimated on the data, the translations of the marker coding may have an additional indirect effect of changing the penalty factors. Also there may be rounding effects impacting the variance component estimates when the entries of the corresponding matrix are too big, and there may be numerical issues related to the matrix inversion when solving for the effects (analogous to Equation (5)). A high dimension or a small determinant of the matrix to be inverted can cause numerical unprecisions which may impact the results. If possible these superposed effects should be separated from each other. For this reason, we follow an approach of reducing the number of variables and estimating the variance components only once and then fixing the penalty factors.

For the considered data set with 1279 markers we deal with 817281 interaction effects when the full model with all pairwise interactions is used. However, if we reduce the number of markers below the number of individuals (599), we can estimate the additive effects as fixed effects and penalize only their interactions. We restrict our considerations to models including 50, 100 or 150 markers and their 1225, 4950 or 11175 interactions, respectively.

For each environment we choose randomly 50 (100, 150) markers using the sample function of R. The eRRBLUP model which we apply afterward includes the fixed effect *μ*, the 50 (100, 150) additive effects and their 1225 (4950, 11175) interactions. The results reported will be based on 50 repeated random draws of the corresponding number of markers. Moreover, we estimate the variance components only for the column mean centered coding and use the corresponding penalty factors also for the estimation with other codings. This is analogous to the fact that the translational invariance of RRBLUP holds when the penalty factor remains fixed (which should be the case when *restricted maximum likelihood* is used for variance component estimation). For the estimation of the variance components, we use the regress package (Clifford *et al.* 2014). The resulting three variance components define the penalty factors. The effects are estimated by the extension of Equation (5) with two different penalty factors for additive effects and interactions. For each of the environments, we compare the Pearson correlation of the estimated interaction effects for the three different codings for 50 randomly drawn sets of markers.

#### Interaction effect estimates and changes in training set size:

The correlation of the interaction effect estimates of eRRBLUP with different codings may not only depend on the number of interactions included, but also on the number of data points provided by y, which means here the number of lines. To compare the effect of an increase in the number of markers to the effect of a reduction of lines used to estimate the interactions, we also compare the effect estimates of different codings, when the number of lines is reduced to 300 or 200. For both sizes, 50 randomly and independently drawn training and marker sets are the basis for interaction effect estimates and their Pearson correlations. The impact of the reduction of lines is only evaluated in the scenario including 50 markers and their interactions.

#### Regressions compared:

In a first scenario, the size of *μ* is not penalized, but the sizes of additive effects and of interactions are. Here, the three variance components $\sigma_{\epsilon }^{2}$, $\sigma_{\beta }^{2}$ and $\sigma_{h}^{2}$ are estimated which define the penalty factors $\lambda_{1}$ for additive effects and $\lambda_{2}$ for the interactions. In a second setup, we only penalize the interaction effects. Here, the additive effects are modeled as being fixed ($\lambda_{1}=0$) and thus, the two variance components $\sigma_{\epsilon }^{2}$, and $\sigma_{h}^{2}$ are estimated and define the penalty factor $\lambda_{2}$ for the interactions. This regression approach is a practical application of Corollary 2 which makes the predictions of genetic values and interaction effects independent from the choice of coding. Thus, from a theoretical perspective, it is clear that the estimates should be identical for the different codings. Yet, we use this second scenario to asses the impact of computational issues –for instance related to the inversion of the corresponding high-dimensional matrix when solving for the interaction effects– on the estimates of interactions.

#### Determining (un)explained phenotypic variance:

To check the influence of the coding on how much phenotypic variance can be explained by the interaction effects, we estimate the variance components on the full data set with all markers and all lines. Since some variance may be attributed “randomly” to additive or epistatic variance, when both corresponding covariance matrices are too similar, we focus on a model with only interaction effects and consider the variance component of the residual, that is the variance which is not explained. The residual variance is more comparable, since the different epistatic relationship matrices may be scaled differently which directly translates to the estimated variance components. We use the equation

$$H=0.5\left({MM}^{t}\circ MM^{t}\right)-0.5\left(M\circ M\right){\left(M\circ M\right)}^{t}$$ (11)

to calculate the epistatic relationship for all 599 lines (Martini *et al.* 2016) and divide by the maximum of H. The regress package (Clifford *et al.* 2014) is used and the residual variance is then compared between different codings. The symbol ∘ denotes Hadamard product, that is the entry-wise multiplication.

#### Out of sample predictions with varying number of markers and training set size:

To assess the impact of translations of the marker coding on out-of-sample predictions $\hat{y}$, we randomly draw test sets of 60 out of the 599 lines to be predicted by the additive and interaction effects estimated from the training set consisting of the remaining 539 lines. We compare the Pearson correlations between the predictions ${\hat{y}}_{test}$ for different codings and with the known phenotypes $y_{test}$ (“predictive ability”). We do this for models including 50, 100 or 150 marker and their interactions. Moreover, we compare the predictions of a model with 50 markers, when the training set size is reduced. Since the variance of the results is increased due to sampling of markers and training and test sets, we use 200 repetitions with independently drawn marker, training and test sets.

### Data Availability

The authors affirm that all data necessary for confirming the conclusions of the article are present within the article, figures, and tables or publicly available (Crossa *et al.* 2010).

### Results

#### Correlation of interaction effect estimates under different codings and varying number of variables included in the model:

We use a model with 50, 100 or 150 markers and their interaction effects and compare the Pearson correlations of the interaction estimates obtained with different codings. The results are summarized in Table 2. The values indicate the mean correlation of 50 randomly drawn marker sets. The correlations are between 0.80 and 0.95 and thus relatively high, but not equal to 1. Moreover, for each environment, and for each comparison of two codings, the correlation of the estimates reduces when the number of markers increases. For instance, an obtained mean correlation of the interaction effect estimates using the originally provided 0,1 coding and the allele-frequency centered coding is 0.90 (environment 2; 50 randomly selected markers). For a model with 100 markers, this correlation reduces to 0.85, and to 0.80 when using 150 markers. This stepwise reduction of the correlation of the estimates when the number of markers increases, can be observed for any pairwise comparison of two codings, and any underlying data (grown in environment 1−4).

**Table 2:** Pearson correlation of the estimates of the 1225 (4950, 11175) interactions when different marker codings are used. The numbers represent the mean correlation of 50 repetitions with independently, uniformly drawn markers. The standard error of the estimate was in all cases below 0.004. Colors indicate the underlying data: Environment 1, 2, 3 or 4.

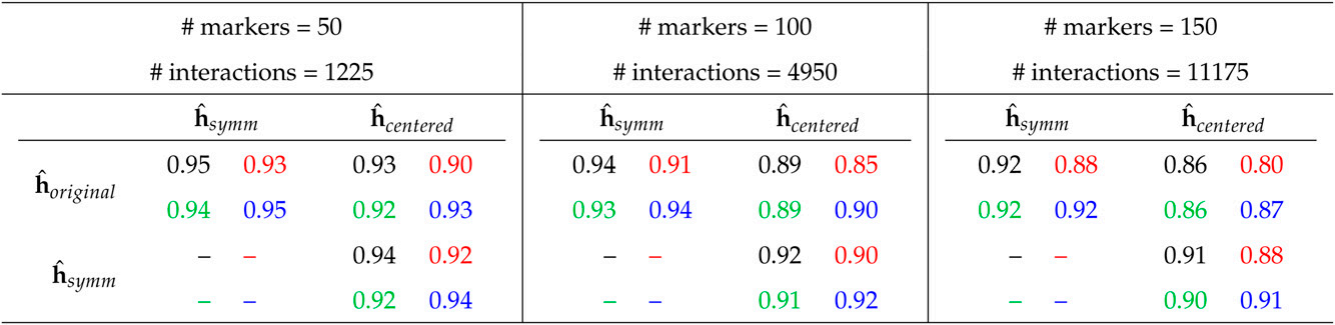

Another observation across environments and varying number of markers is that the original 0,1 coding and the column mean centered coding are the most different. For instance, for environment 2 and 150 marker, the mean correlation of the interaction effect estimates is 0.80, which is smaller than the corresponding correlation for original and symmetric coding (0.88) or for symmetric and centered coding (0.88).

For the second type of regression, in which only the size of the interactions are penalized, we receive –as stated by our theoretical results– a correlation of 1 between any two codings, for each of the four underlying environmental conditions and for each number of considered markers. This circumstance also illustrates that potential numerical problems –for instance related to the inversion of the high-dimensional matrix of Eq.(5)– do not strongly occur with the number of interactions modeled in our examples.

#### Ranks of interactions effects:

The previously described observations have been made on the level of correlations of interaction effect estimates. To address the question of what they mean for an individual interaction effect estimate, we consider an example of using the 150 markers with largest additive effects (Kärkkäinen *et al.* 2015). We are interested in changes of interaction effect sizes and in their relative importance. We rank the estimated interactions according to their absolute values and compare these rankings for the different codings. The maximal observed rank change of an estimate is 10367 for the data of environment 1, with an interaction that is the 362^th^ highest when the original 0,1 coding is used, but with a rank of 10729 with the centered coding. Analogously, the maximal observed rank changes are for environments 2 to 4, 10775, 10672 and 10750, respectively. All maximal rank changes are observed comparing the original and the centered coding. Recall here that there are only 11175 interactions considered. The maximal theoretically possible rank change would thus be 11174. The relative importance of these interactions changes from the top 4% to the 4% most unimportant interactions. Their effects are lost when we translate from 0,1 to the centered coding.

#### Reducing training set size:

The increasing influence of the choice of coding on interaction effect estimates when the number of interactions is increasing, may have a counterpart when the training set size is decreasing. We use a model including 50 randomly chosen markers and their interactions and reduce the training set size from 539 to 300 or to 200. Table 3 presents the mean Pearson correlations of the interaction estimates based on 50 randomly drawn marker and training sets (of 200 or 300 lines, respectively). Here, the reduction in correlation has a similar pattern to the situation in which the number of markers in increasing. However, comparing the situation of 150 markers and 599 lines (Table 2) to the situation of 50 markers and 200 lines (Table 3), we see that the impact of coding on interaction effect estimates is more strongly affected by the reduction in training set size than by the increase in the number of markers. For instance, the lowest correlation in Table 2 is 0.80 for 150 markers and the data of environment 2. In Table 3, the correlation with a test set size of 200 is 0.68. In particular, this shows that the impact does not only depend on the ratio of training set size and markers.

**Table 3:** Pearson correlation of the estimates of the 1225 interaction effects when different marker codings are used and the number of genotypes used to estimate the effects is reduced from the 599 available lines to 300 or 200, respectively. Only the model with 50 markers and 1225 interactions is considered. The numbers represent the mean correlation of 50 repetitions with independently, uniformly drawn markers and lines. The standard error of the estimate was in all cases below 0.025. Colors indicate the underlying data: Environment 1, 2, 3 or 4.

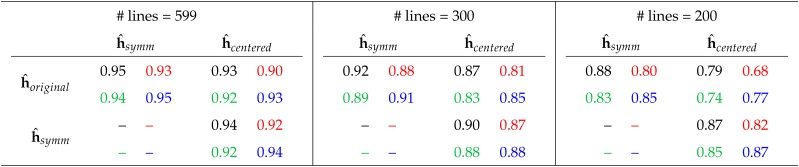

#### (Un)explained variance:

To illustrate which impact the coding may have on the phenotypic variance an epistatic relationship matrix can capture, we use only the epistatic relationship to estimate a corresponding variance component and a residual variance. In a model with additive and epistatic relationship, some of the phenotypic variance may be explained by either one or the other. Thus, if the relationship matrices are not very different, small details on the phenotypic data can determine whether the variance is more attributed to the additive or to the epistatic part. If we only use the epistatic part, we can better highlight the changes in the phenotypic variance that may be explained by the epistatic relationship in the respective coding. The results are illustrated in Table 4. Whereas for the additive matrix the residual variance estimate is constant across the three different codings (not shown), the estimated residual variance varies strongly when the epistatic relationship matrix is constructed with different codings. The range of residual variance is between 0.33 and 0.42, 0.46 and 0.53, 0.35 and 0.54, and 0.38 and 0.48, for environments 1−4, respectively. On average, the symmetric coding shows the lowest unexplained variance.

**Table 4:** **Unexplained variance: Residual variance** $\sigma_{\epsilon }^{2}$ **estimated with epistatic relationships based on the three different codings and for the four different environments. The standard error returned by regress() was for each estimated residual variance between 0.045 and 0.064.**

	Env1	Env2	Env3	Env4
original	0.42	0.51	0.54	0.48
symm	0.33	0.46	0.40	0.38
centered	0.36	0.53	0.35	0.40

#### Out-of-sample predictions under varying number of variables included in the model:

Analogously to the experiment of increasing the number of variables in the model and comparing the interaction effect estimates, we also compare “out-of-sample” predictions. As previously described, eRRBLUP models based on 50, 100 or 150 markers are used to predict a test set of 60 lines by the effects estimated from the remaining 539 lines. The results for 200 randomly drawn test and marker sets are summarized in Table 5. An (expected) observation is that the predictive ability increases with the number of markers. For instance for environment 1, the predictive ability increases from 0.44 to 0.49 for the original coding, and from 0.42 to 0.49 for the symmetric coding. This observation is consistent across all coding-environment combinations.

**Table 5:** Pearson correlation of predictions of a test set consisting of 60 lines when predicted by additive and interaction effect estimates based on a model including 50, 100 or 150 markers and their pairwise interaction effects. The numbers represent the means of 200 repetitions with independently, uniformly drawn marker and test sets. The training set is given by the remaining 539 lines. The standard error of the estimate was in all cases smaller than 0.01. Colors indicate the underlying data: Environment 1, 2, 3 or 4.

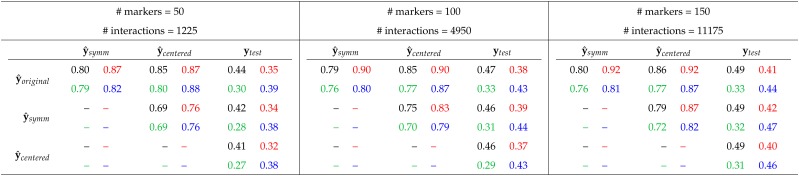

Moreover, contrarily to the observation we have made for the interaction effect estimates for which the correlations reduce when more markers are included into the model, the correlations of “out-of-sample” predictions $\hat{y}$ do not show this pattern. The results rather suggest that there is an increase in the correlation when more markers are included. For instance, with the data of environment 1, the correlation between ${\hat{y}}_{symm}$ and ${\hat{y}}_{centered}$ increases from 0.69 to 0.79 from 50 to 150 markers. This observation is not consistent across all coding-environment combinations, but an increasing correlation with the number of markers is the more prevalent case. Overall, we see that in 7 out of 12 cases, an increasing number of markers leads to an increase in correlation between different predictions. In one case –${\hat{y}}_{original}$ and ${\hat{y}}_{symm}$ for environment 1– no change is observed, and in four cases, the correlation of the predictions is reduced.

#### Out-of-sample predictions under varying training set size:

Analogously to having reduced the training set size when we have investigated the correlation of interaction effect estimates, we also perform a similar experiment for “out-of-sample” prediction. The results are summarized in Table 6. An obvious and clear pattern is the reduction in predictive ability when the training set size is reduced for each coding-environment combination. Similar to the situation of increasing the number of interactions modeled, there is no clear pattern visible for the correlations of predictions obtained with different codings.

**Table 6:** **Pearson correlation of predictions of a test set consisting of 60 lines when predicted by additive and interaction effect estimates based on a model including 50 randomly chosen markers and their pairwise interaction effects. The numbers represent the means of 200 repetitions with independently, uniformly drawn marker and test and training sets (The latter of size 539, 300 or 200). The standard error was in all cases smaller than 0.022. Colors indicate the underlying data: Environment 1, 2, 3 or 4. The case of a training set size of 539 is the case of 50 markers in** **Table 5**.

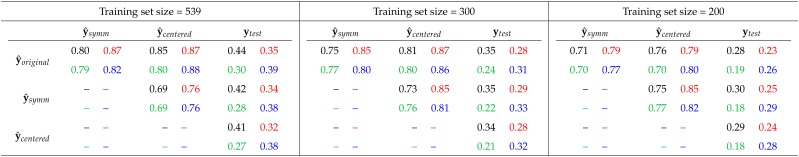

## Discussion

### Interpretability of effect estimates

The illustrated problem of the coding having an impact on the estimates of interactions in penalized regressions is essential for quantitative genetics, where Hadamard products are often used to model interaction such as epistasis or gene by environment interaction (Pérez-Rodríguez *et al.* 2017; De Coninck *et al.* 2016; Shang *et al.* 2015; Sukumaran *et al.* 2017). Hadamard products of covariance matrices represent exact reformulations of certain interaction effect models (Jiang and Reif 2015; Martini *et al.* 2016).

In particular, our observations illustrate once more that the size of interaction effect estimates obtained from WGR should be interpreted with caution because a biological meaning is not necessarily given. On the one hand the data structure (*e.g.*, population stratification), and on the other hand the coding may influence effect estimates. In models penalizing interactions and additive effects, the coding issue alone can have a drastic impact on the interaction estimates as illustrated by the consideration of the ranks of interaction effect. Some interactions were among the 4% with the highest absolute interaction effect with one coding and among the 4% with the lowest absolute effects with another coding. Moreover, as illustrated in Table 2, the impact of variable coding on interaction effect estimates, increases with the number of modeled interactions. This observation may not be surprising, since with more interactions there are more changes when the coding is altered, and a higher number of variables also provides more flexibility to model certain effects by other interactions. However, this circumstance illustrates that the supposed advantage of an increased interpretability when modeling epistasis effects explicitly instead of “hidden” in an RKHS approach, may only be marginal. This statement does not doubt that the overall predictive ability for y may be increased when epistasis models are used (which has been demonstrated for instance by Ober *et al.* (2015), Jiang and Reif (2015), Martini *et al.* (2016)). In particular, interaction effects estimates have been used to select important interactions and thus improving predictive ability for different environment conditions by reducing the model to the “more relevant” interactions (Martini *et al.* 2016). However, the biological meaning of individual interaction effects obtained from an epistatic WGR is limited (at currently used training set sizes). In this regard, Corollary 2 illustrated that approaches selecting markers first and then modeling the interactions between them (Kärkkäinen *et al.* 2015) may provide the option to model additive effects as being fixed and to only penalize interactions effects, thus at least eliminating the coding problem and potentially facilitating the attempt to assign a biological meaning to estimated quantities. However, this raises the question of how to select the variables. Using the additive effect size as a selection criterion might for instance have the danger of missing relevant interactions, since some pairs of loci could have small additive effects but potentially large interaction effects (Mackay and Moore 2014).

### Impact of coding on out-of-sample predictions

Interestingly, the observed effect of a decreasing correlation of interaction effect estimates when the number of markers is increasing cannot be observed for the correlation of out-of-sample predictions $\hat{y}$. The different behavior of the correlations of interaction effect estimates (Table 2) and out-of-sample predictions (Table 5) illustrates that different WGRs may model the sum of their effects similarly, but not necessarily each summand. The estimates from a global, joint consideration of all markers will give a good description of the sum of the effects, but not necessarily of each effect. To increase the biological meaning of interaction effect estimates obtained from epistatic WGR, training set sizes will have to increase drastically to make the data more and the prior assumption less important.

### Is there a better and a worse coding?

Out of the three codings compared here, the symmetric coding explains the phenotypic variation best on the considered data set (Table 4). In the case of using only the epistatic relationship, this has also been reflected by showing the highest predictive ability when all markers were used (Martini *et al.* 2017). Moreover, Santantonio *et al.* (2018) also observed a slightly improved predictive ability for the symmetric coding on a different data set. In this work, in which we modeled additive and epistatic effects, and restricted the number of markers, no clear superiority of one or the other coding was observed. The results for the case of 150 markers presented in Table 5, might be interpreted as a sign that for a higher number of markers, the symmetric coding leads to the higher predictive ability. However, these observations do not provide a theoretical explanation for why a certain coding should be better than the other. An earlier theoretical explanation was that the symmetric coding has the advantage of being independent of the choice of the reference allele. Since other codings are affected by the choice of which allele is set as the reference, additional uncertainties may be introduced. The symmetric coding does not have this additional problem, since changing the reference allele does not have an impact on the size of the effect estimate, but only on its sign (Martini *et al.* 2017).

What certainly has an impact on predictive ability, when several covariance matrices are used, is how “similar” the matrices are. A good coding should make the covariance matrices more “different” to allow them to capture different components of the phenotypic variance. It is important to note here that this question may sound similar to the question of orthogonal effect estimate coding, but the level aimed at is different. Applying the coding proposed by Vitezica *et al.* (2017) does not necessarily make the additive G and the epistatic relationship G∘G orthogonal to each other.

In this regard, we also highlight that we investigated the importance of coding with respect to the method applied. In particular, the OLS approach applied to a model with interactions, but also a penalized regression only penalizing the effect sizes of monomials of highest total degree, will both provide predictions $\hat{y}$, as well as estimated effect sizes of monomials of highest total degree, which are independent of the coding. For these methods, these estimates will not depend on whether an “orthogonal” coding is used. We did not consider how estimates of dominance effects may relate to the estimates of additive effects or other analogous relations. Contrarily, the topic of orthogonal estimates has been discussed independently of the method applied afterward, but more in the context of how the different effects relate to each other. Thus, both discussions do not coincide.

### Epistatic effect models and the Gaussian kernel

Finally, note that it has been reported that a Gaussian reproducing kernel Hilbert space regression (Morota and Gianola 2014) can be interpreted as a limit of a penalized polynomial regression with increasing total degree (and all possible monomials) (Jiang and Reif 2015). Being a limit case of a method which is affected by translations of the coding, the question appears why the Gaussian kernel regression is invariant to translations of the marker coding (The invariance of the Gaussian kernel is a direct consequence of being defined on the Euclidean distance. If two genotypes are translated in the same way, their distance remains unchanged). It may be interesting from a theoretical point of view to reconsider the limit behavior of polynomial regression.

### Summary

We identified the cause of the coding-dependent performance of epistasis effect models. Our results were motivated by ridge regression, but do equally hold for many other types of penalized regressions, for instance for the $\ell_{1}$ penalized LASSO. The fact that the estimated effect sizes depend on the coding highlights once more that estimated interaction effect sizes should be interpreted with caution with regard to their biological, mechanistic meaning. In particular, the supposed advantage of a facilitated interpretability compared to RKHS methods may not be given when epistatic whole genome regressions are used. Moreover, the problem of coding is not only present for marker by marker interaction, but for any mixed model in which interactions are modeled by Hadamard products of covariance matrices, in particular also for gene by environment (G x E) models.

### Outlook

The work on hand only addressed coding translations, but not scaling of markers. It is clear that scaling has an impact on effect estimates due to changing the penalty factors individually. How to scale markers optimally in an additive effect model is not completely understood and this investigation may also be extended to the situation of epistasis models. Moreover, the question on how to make the additive and interaction effect matrix most different should be addressed in the future.

## Appendix: Proofs

*Proposition 1*. The fact that the SSR remains the same, results from the definition of the polynomials. To see that the coefficients of monomials of highest total degree D are identical, choose a monomial $m\left(M_{l_{1}},M_{l_{2}},\ldots ,M_{l_{d}}\right)$ of the loci $l_{1},\ldots ,l_{d}$ of total degree *D* of *f*. Expanding $m\left({\tilde{M}}_{l_{1}}+P_{l_{1}},{\tilde{M}}_{l_{2}}+P_{l_{2}},\ldots ,{\tilde{M}}_{l_{d}}+P_{l_{d}}\right)$ gives the same monomial $m\left({\tilde{M}}_{l_{1}},{\tilde{M}}_{l_{2}},\ldots ,{\tilde{M}}_{l_{d}}\right)$ as a summand of highest total degree, plus additional monomials of lower total degree. Thus, the coefficients of monomials of total degree *D* remain the same. ∎

*Corollary 1*. Let ℱ be the set of polynomials in the variables ${\left(M_{i,j}\right)}_{j=1,\ldots ,p}$ across which we look for the one minimizing the SSR with respect to our data (the OLS fit). Let $\tilde{ℱ}$ be the polynomials in the variables ${\left({\tilde{M}}_{i,j}\right)}_{j=1,\ldots ,p}$. Given the vector $P^{t}$, which defines the alternative coding by $\tilde{M}:=M-1_{n}P^{t}$, the definition of $\tilde{f}$ in Proposition 1 defines a map

$$T:ℱ\to \tilde{ℱ}$$

$$T\left(f\left(M_{i,•}\right)\right)=\tilde{f}\left({\tilde{M}}_{i,•}\right):=f\left({\tilde{M}}_{i,•}+P^{t}\right).$$

This equation states that $\tilde{f}$ which is a polynomial in the variables ${\tilde{M}}_{i,•}$ is defined by the original *f* when we plug in the variables ${\tilde{M}}_{i,•}+P^{t}$ and write down the expression as a function of ${\tilde{M}}_{i,•}$. The SSR can be considered as a function

$$\text{SSR}:ℱ\to {\mathbb{R}}_{0}^{+}$$

f↦SSR(f,y,M).

Since *f* and $\tilde{f}$ fit the data in the same way, we have $\text{SSR}\left(f,y,M\right)=\text{SSR}\left(\tilde{f},y,\tilde{M}\right)$. Thus, for a solution $f_{0}$ minimizing the SSR across all polynomials in coding M, ${\tilde{f}}_{0}$ minimizes the SSR in coding $\tilde{M}$.∎

*Corollary 2*. Analogously to the proof of Corollary 1, but with a function SSR${}_{2}$, which is the sum of SSR and the penalty on the coefficients of monomials of highest degree, which are the same for *f* and $\tilde{f}$, which implies ${\text{SSR}}_{2}\left(f,y,M\right)={\text{SSR}}_{2}\left(\tilde{f},y,\tilde{M}\right)$.∎

*Corollary 3*. A special case of Corollary 2, since only the additive effects, that is only the coefficents of monomials of highest total degree are penalized.∎

*Corollary 4*. Analogous to Corollary 3.∎
